# Preparation of Risedronate Nanoparticles by Solvent Evaporation Technique

**DOI:** 10.3390/molecules191117848

**Published:** 2014-11-04

**Authors:** Eliska Vaculikova, Daniela Placha, Martin Pisarcik, Pavlina Peikertova, Katerina Dedkova, Ferdinand Devinsky, Josef Jampilek

**Affiliations:** 1 Department of Chemical Drugs, Faculty of Pharmacy, University of Veterinary and Pharmaceutical Sciences, Palackeho 1/3, Brno 61242, Czech Republic; 2 Nanotechnology Centre, VSB—Technical University of Ostrava, 17. listopadu 15/2172, Ostrava 70833, Czech Republic; 3 Department of Chemical Theory of Drugs, Faculty of Pharmacy, Comenius University, Kalinciakova 8, Bratislava 83232, Slovakia

**Keywords:** risedronate sodium, nanoparticles, excipients, solvent evaporation, dynamic light scattering, infrared spectroscopy, scanning electron microscopy

## Abstract

One approach for the enhancement of oral drug bioavailability is the technique of nanoparticle preparation. Risedronate sodium (Biopharmaceutical Classification System Class III) was chosen as a model compound with high water solubility and low intestinal permeability. Eighteen samples of risedronate sodium were prepared by the solvent evaporation technique with sodium dodecyl sulfate, polysorbate, macrogol, sodium carboxymethyl cellulose and sodium carboxymethyl dextran as nanoparticle stabilizers applied in three concentrations. The prepared samples were characterized by dynamic light scattering and scanning electron microscopy. Fourier transform mid-infrared spectroscopy was used for verification of the composition of the samples. The particle size of sixteen samples was less than 200 nm. Polysorbate, sodium carboxymethyl dextran and macrogol were determined as the most favourable excipients; the particle size of the samples of risedronate with these excipients ranged from 2.8 to 10.5 nm.

## 1. Introduction

The bioavailability of drugs is influenced by their solubility and permeability. When per-oral administered drugs display good water solubility, permeability is a critical issue [1,2]. In the Biopharmaceutical Classification System (BCS) high water solubility is defined as: (a) 85% dissolution of the dose within 30 min at all pH values from 1 to 7.5 and (b) dose/solubility ≤250 mL. The difference between a high-solubility and a low-solubility compound can be one million-fold (0.1 µg/mL–100 mg/mL). A drug substance is considered highly intestinal permeable if >90% of the administered drug dose is absorbed in comparison with intravenous administration. The difference between a high-permeability and a low-permeability compound can be 50-fold (0.001–0.05 min^−1^) [3]. The permeability of orally dosed drugs depends on several factors such as intestinal permeability, solubility in gastrointestinal system, drug release from the dosage form, liability to efflux and metabolism. Generally strategies/structural modifications to improve permeability are based on a few fundamental concepts: reduction of ionizability, increase of lipophilicity, reduction of polarity or reduction of hydrogen bond donors or acceptors. Formulation is other strategy for improving permeability and bioavailability; for example, permeability enhancers, surfactants or pharmaceutical complexing agents can be used in the oral dosage form [3]. The problem of poor permeability can be also solved by preparation of nanoparticles. In general, nanoparticles for systemic applications should range from 10 to 100 nm in size and have minimum surface charge [4]. A lot of nano-based permeability enhancing techniques is known, e.g., preparation of nano-emulsions using excipients with solubilizing or permeation enhancing properties [5,6,7]. Nanosize allows effective systemic circulation and enables many pharmacological agents to reach sites of action that are not available to larger particles [8,9,10,11].

**Figure 1:**
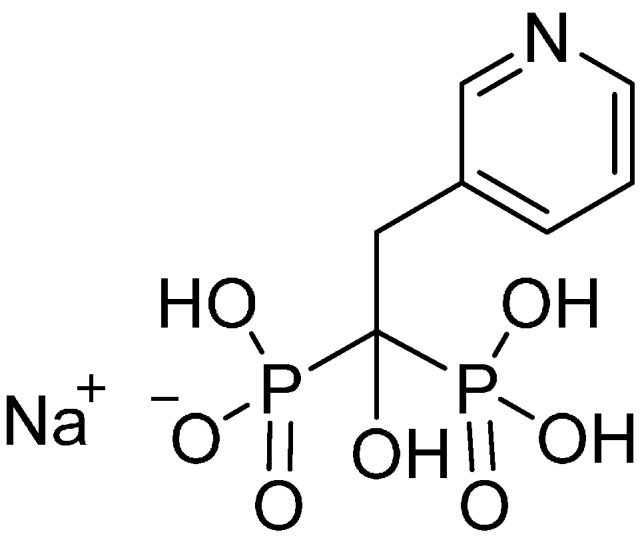
Structure of sodium salt of risedronate.

Bisphosphonates (BPs) represent highly hydrophilic compounds with low oral bioavailability (in most cases less than 3%) [12]. These pyrophosphate analogues are the most effective bone resorption inhibitors [13]. BPs contain a P-C-P backbone with two covalently bound side chains by which BPs differ from each other. The mechanism of action of BPs is induction of apoptosis in osteoclasts [14]. Risedronate (risedronate monosodium salt, systematic name: sodium 1-hydroxy-1-phosphono-2-(pyridin-3-yl-ethyl)phosphonate, Figure 1), is a member of Class III of the BCS and belongs to the third-generation BPs. It has a chemically unique component as compared with other BPs, which is believed to reduce the likelihood of gastro-intestinal side effects. Risedronate is more potent in blocking the dissolution of bone than other BPs [15,16]. It has good solubility in water, but its absolute bioavailability after oral administration is less than 1% [17,18]. Due to these facts risedronate was chosen as a model drug in this study.

BPs are used as per-oral or injection forms, nevertheless new formulations of BPs are developed with a view to increase their oral bioavailability and reduce side effects. For example, the use of ethylenediaminetetraacetic acid in BP formulations enhanced their absorption in intestine [19]. Other approaches to the modification of BP properties are the preparation of co-crystals [20,21] or the preparation of chitosan-coated mucoadhesive liposomes or peptide prodrugs that can be recognized by the intestine carrier system and subsequently transported. Also, an adduct of risedronate with titanium dioxide particles was proposed as a controlled-release system [22].

This contribution is a follow-up paper to our previous studies [20,21,23,24,25] and deals with preparation of nanoparticles of risedronate monosodium salt by means of the solvent evaporation method. A solution of excipient is mixed under continuous stirring with a solution of the drug substance and then the second solvent is evaporated. An excellent review dealing with this technique was published by Thorat *et al.* [26]. Nanoparticles are stabilized by various excipients selected based on the Generally Recognized as Safe (GRAS) list [27], which means that they are not toxic to the human body. The excipients were applied in various concentrations, because the optimal concentration of surfactant is important for optimal particles wetting. If the concentration is too low, particles float on the surface. If the concentration is too high, bubbles appear [28]. The particle size of all the prepared samples was analysed by means of dynamic light scattering. The samples were also characterized by means of scanning electron microscopy (SEM), and the composition was verified by Fourier transform mid infrared (FT-MIR) spectroscopy.

## 2. Results and Discussion

Based on pilot screening [23,24,25], five different excipients and one mixture of excipients were used in this investigation. The used excipients represent various classes of low-molecular or polymeric pharmaceutical adjuvants (non-ionic or anionic surfactants, emulsifiers/viscosity modifiers/thickeners) that can be utilized as solubility modifying compounds/nanoparticle stabilizers. They included sodium dodecyl sulfate (SDS, Sample series **1**), polysorbate 80 (PLS, Sample series **2**), macrogol 6000 (PEG, Sample series **3**), sodium carboxymethyl cellulose (SCMC, Sample series **4**), sodium carboxymethyl dextran (SCMD, Sample series **5**) and sodium dodecyl sulfate/macrogol 6000 (DSP, Sample series **6**). Three water solutions were prepared from each excipient with mass concentration of 1% (Samples **a**), 3% (Samples **b**) and 5% (Samples **c**). As risedronate sodium salt is soluble in water, this medium was chosen as a solvent. Risedronate sodium was dissolved in water and added to the solution of excipient under continuous stirring. Then an ultrasonic bath was used for destruction of possible agglomeration, and finally the solvent was evaporated. Combination of all excipients with risedronate provided eighteen samples, see Table 1.

**Table 1:** Composition of samples, concentration (%) of individual excipients in water samples relative to risedronate, particle size (nm) and polydispersity index (PDI) of risedronate samples expressed as mean ± SD (*n* = 5 independent measurements). (SDS = sodium dodecyl sulfate, PLS = polysorbate 80, PEG = macrogol 6000, SCMC = sodium carboxymethyl cellulose, SCMD = sodium carboxymethyl dextran, DSP = sodium dodecyl sulfate/macrogol 6000).

Sample	Excipient/Concentration (%)	Particle Size (nm)	PDI
**1a**	SDS/1	95.6 ± 11.2	0.263 ± 0.030
**1b**	SDS/3	194.8 ± 34.6	0.377 ± 0.056
**1c**	SDS/5	75.4 ± 3.4	0.285 ± 0.022
**2a**	PLS/1	10.5 ± 0.3	0.173 ± 0.048
**2b**	PLS/3	10.1 ± 0.3	0.086 ± 0.043
**2c**	PLS/5	9.9 ± 0.1	0.139 ± 0.017
**3a**	PEG/1	9.1 ± 1.9	0.532 ± 0.095
**3b**	PEG/3	8.2 ± 1.1	0.401 ± 0.066
**3c**	PEG/5	7.7 ± 2.5	0.315 ± 0.028
**4a**	SCMC/1	12.9 ± 2.4	0.685 ± 0.101
**4b**	SCMC/3	2946.7 ± 367.6	0.560 ± 0.048
**4c**	SCMC/5	7287.6 ± 1352.5	0.651 ± 0.151
**5a**	SCMD/1	3.5 ± 1.1	0.473 ± 0.156
**5b**	SCMD/3	2.8 ± 0.4	0.287 ± 0.050
**5c**	SCMD/5	3.8 ± 1.2	0.244 ± 0.135
**6a**	DSP/1	110.1 ± 7.4	0.578 ± 0.029
**6b**	DSP/3	122.7 ± 7.4	0.524 ± 0.037
**6c**	DSP/5	4.00 ± 0.8	0.310 ± 0.033

**Figure 2:**
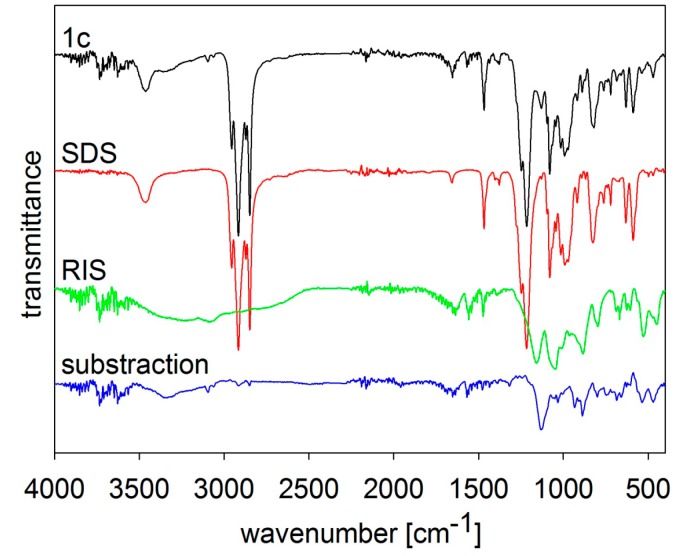
MIR spectra of risedronate (RIS), sodium dodecyl sulfate (SDS) and risedronate nanoparticles stabilized with 5% concentration of sodium dodecyl sulfate (Sample **1c**) and differential spectrum.

**Figure 3:**
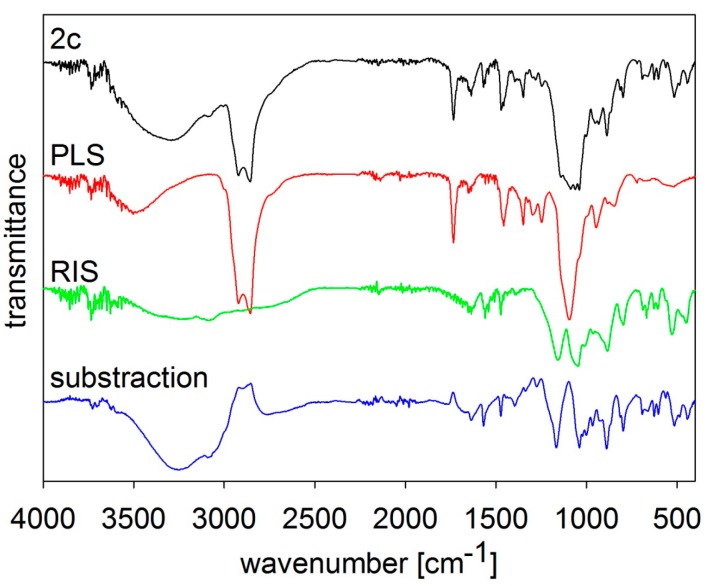
MIR spectra of risedronate (RIS), polysorbate 80 (PLS) and risedronate nanoparticles stabilized with 5% concentration of polysorbate 80 (Sample **2c**) and differential spectrum.

**Figure 4:**
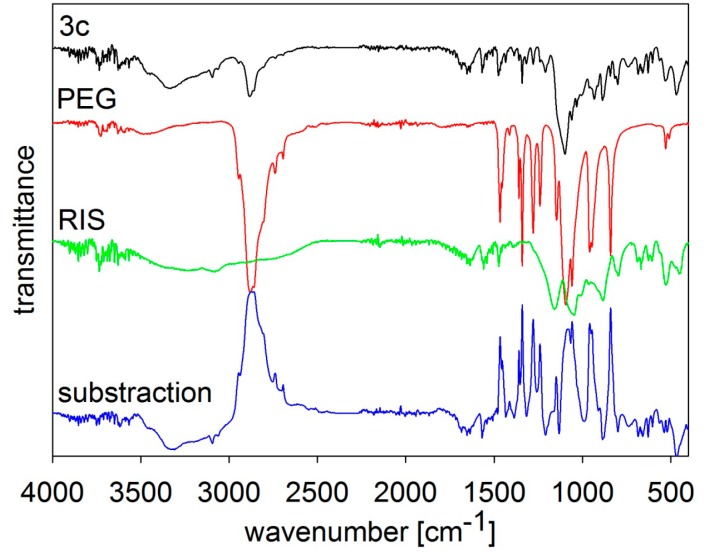
MIR spectra of risedronate (RIS), macrogol 6000 (PEG) and risedronate nanoparticles stabilized with 5% concentration of macrogol 6000 (Sample **3c**) and differential spectrum.

**Figure 5:**
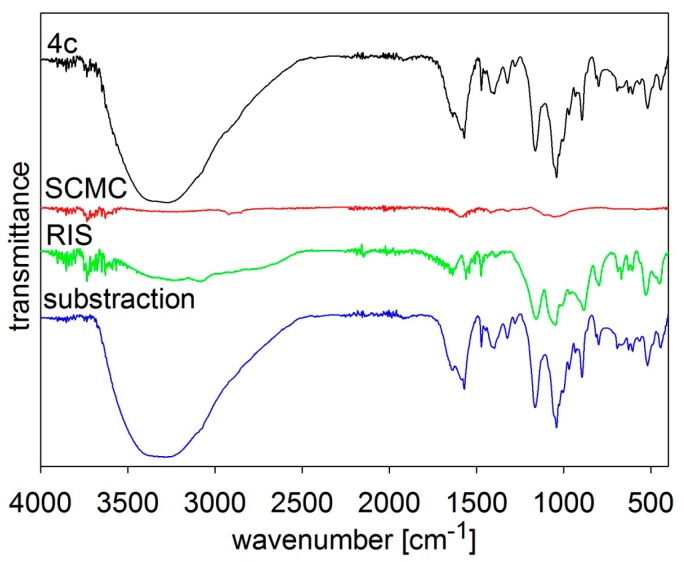
MIR spectra of risedronate (RIS), sodium carboxymethyl cellulose (SCMC) and risedronate nanoparticles stabilized with 5% concentration of sodium carboxymethyl cellulose (Sample **4c**) and differential spectrum.

**Figure 6:**
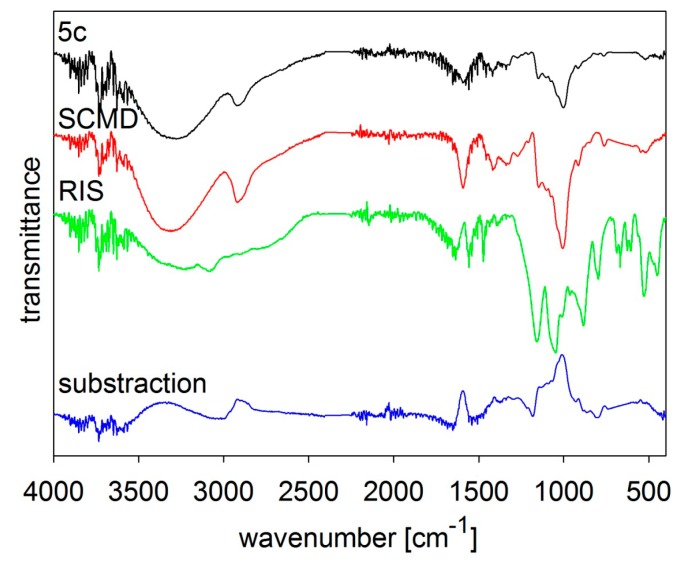
MIR spectra of risedronate (RIS), sodium carboxymethyl dextran (SCMD) and risedronate nanoparticles stabilized with 5% concentration of sodium carboxymethyl dextran (Sample **5c**) and differential spectrum.

The composition of the samples was verified by FT-MIR spectroscopy, see Figure 2, Figure 3, Figure 4, Figure 5 and Figure 6. The individual figures illustrate spectra of the starting materials and the product. Also differential spectra (product-excipient) are illustrated to confirm the presence of risedronate and to verify possible interactions between risedronate and the excipient in the sample. No interactions were found, *i.e.*, all the samples were simple mixtures, in which risedronate particles were embedded in the excipient.

Figure 2 shows the MIR spectra of risedronate, sodium dodecyl sulfate and risedronate nanoparticles stabilized with sodium dodecyl sulfate (Sample **1c**) and the differential spectrum of sodium dodecyl sulfate and Sample **1c**. The spectrum of Sample **1c** contains all typical absorption bands of risedronate and sodium dodecyl sulfate in the region of 450–1700 cm^−1^ and 2800–3800 cm^−1^. The intensity of the absorption bands of sodium dodecyl sulfate overlapped the spectrum of risedronate. The differential spectrum does not vary significantly; there are no changes in these two structures after preparation process.

The MIR spectra of risedronate, polysorbate 80, risedronate nanoparticles stabilized with polysorbate 80 (Sample **2c**) and the differential spectrum of polysorbate 80 and Sample **2c** are shown in Figure 3. The MIR spectrum of Sample **2c** is composed of absorption bands characterizing both original substances in the region of 450–1750 cm^−1^ and 2800–3800 cm^−1^. There are no significant changes in the differential spectrum and the spectrum of risedronate.

The MIR spectra of risedronate, macrogol 6000 and their mixture (Sample **3c**) and the differential spectrum of macrogol 6000 and Sample **3c** are presented in Figure 4. The spectrum of Sample **3c** contains characteristic absorption bands of both substances as in previous figures.

Figure 5 and Figure 6 illustrate spectra of risedronate and sodium carboxymethyl cellulose and risedronate and sodium carboxymethyl dextran respectively, including their mixtures (Samples **4c** and **5c**), and the differential spectra. There are no interactions between risedronate and excipient in Sample **4c** as observed in the differential spectra in Figure 5. In case of Sample **5c** the spectrum of carboxymethyl dextran overlapped that of risedronate, see Figure 6. All the MIR spectra (Figure 2, Figure 3, Figure 4, Figure 5 and Figure 6) confirmed that Samples **1c**–**5c** contained both risedronate and the excipient and that there were no reactions between these two components.

All prepared samples were measured by dynamic light scattering [29], *i.e.*, particle size and values of polydispersity index were determined. Moreover, samples were characterized by means of SEM, see below. The particle size distribution is presented in Table 1. The investigated particles showed good particle size stability throughout the light scattering measurements. Within the period of measurements, no significant deviations from the mean values of particle size, which could be a result of possible sample ageing, were observed. Also, a regular visual check of the samples proved no changes in the sample structure, which was confirmed by the reproducible data obtained by the light scattering method.

Nanoparticles of size under 200 nm were prepared in the case of sixteen samples. Sodium carboxymethyl cellulose provided micro-size (Samples **4b**, **4c**). Sodium dodecyl sulfate in 3% concentration (Sample **1b**) provided the largest nanoparticles or, more precisely, submicroparticles (*ca.* 195 nm) and in 1% and 5% concentrations (Samples **1a**, **1c**) together with the combination of SDS with PEG in 1% and 3% concentrations (Samples **6a**, **6b**) provided particle size about 100 nm. A significant effect of the applied concentration of the excipient was observed for sodium dodecyl sulfate and the combination of SDS with PEG. The smallest particles were found at 5% concentration of the excipient (Samples **1c**, **6c**). In the case of sodium carboxymethyl cellulose its poor solubility in water plays an important role, and probably, therefore, the most significant effect of SCMC was observed at 1% concentration (Sample **4a**). It is evident that carboxymethyl dextran sodium salt provided slightly smaller nanoparticles (range 2.8–3.5 nm) in comparison with macrogol (7.7–9.1 nm) or with polysorbate (9.9–10.5 nm). According to the results of SCMD, PEG and PLS application (see Table 1), no influence of the concentration of the excipients on the particle size was observed.

The dispersity is a measure/degree of the homogeneity/heterogeneity of sizes of particles in a mixture/system. The uniformity of dispersed systems is expressed as polydispersity index (PDI), see Table 1. Low PDI values demonstrate narrow size distribution and uniformity of samples contrary to PDI ≈ 1 that indicates that samples have a very broad size distribution and may contain large particles or aggregates and are not suitable for measurements [29,30]. In the prepared nanoparticles of risedronate PDI values ranged from 0.086 ± 0.043 to 0.578 ± 0.029, when the samples stabilised by sodium carboxymethyl cellulose (Samples **4a**–**c**) were eliminated. From the results it is evident that the mentioned excipient is not suitable as a nanoparticle stabilizing agent, either for high degree of heterogeneity (Sample **4a**) or for generation of micro-size (Samples **4b**, **4c**). The highest uniformity can be seen for polysorbate, the lowest for sodium carboxymethyl cellulose. The rest of the used excipients showed approximately similar effect on particle size homogeneity.

**Figure 7:**
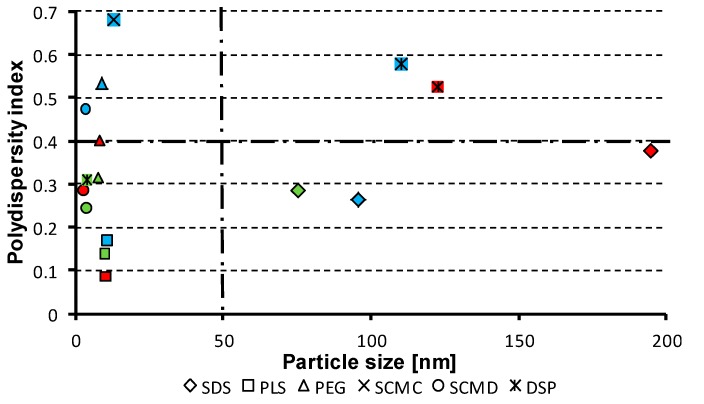
Relationships between polydispersity index values and particle size of risedronate sodium in relation to applied excipients. (SDS = sodium dodecyl sulfate, PLS = polysorbate 80, PEG = macrogol 6000, SCMC = sodium carboxymethyl cellulose, SCMD = sodium carboxymethyl dextran, DSP = sodium dodecyl sulfate/macrogol 6000; fill colour of individual symbols depends on concentration of excipient: 1% = blue, 3% = red, 5% = green).

The dependence of the type and the concentration of the excipient on the particle size and the polydispersity index of individual systems was discussed above. Figure 7 shows the relationship between the polydispersity index values and the particle size of risedronate sodium in relation to the applied excipients. It can be stated that for generation of nanoparticles it is favourable when prepared particles are small, and polydispersity index values are sufficiently low (around 0.5 and less). Based on the results the samples placed in the bottom left quadrant of the graph in Figure 7 meet these requirements. Based on this expectation polysorbate 80 (Samples **2a**–**c**) in all used concentrations seems to be preferable excipient followed by sodium carboxymethyl dextran and macrogol 6000 at 3% and 5% concentrations (Samples **5b**, **5c** and **3b**, **3c**). It can be concluded that the applied method can be used as an effective and affordable technique for preparation of nanoparticles. The selected conditions are convenient for formation of nanoparticles, and the used excipients are principally applicable as nanoparticle stabilizers.

**Figure 8:**
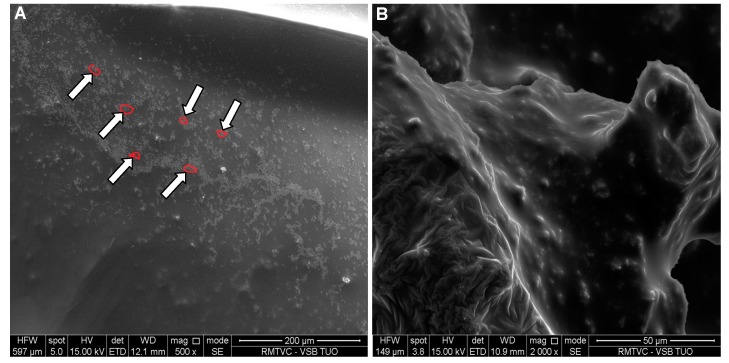
SEM image of risedronate with polysorbate 80 (Sample **2c**) at magnification 500× (**A**) and 2000× (**B**).

**Figure 9:**
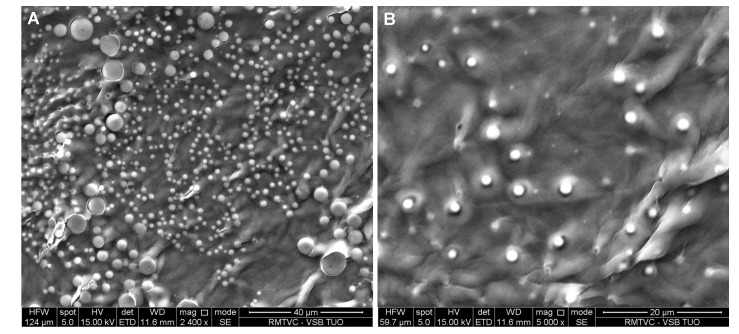
SEM image of risedronate with macrogol 6000 (Sample **3c**) at magnification 2400× (**A**) and 5000× (**B**).

**Figure 10:**
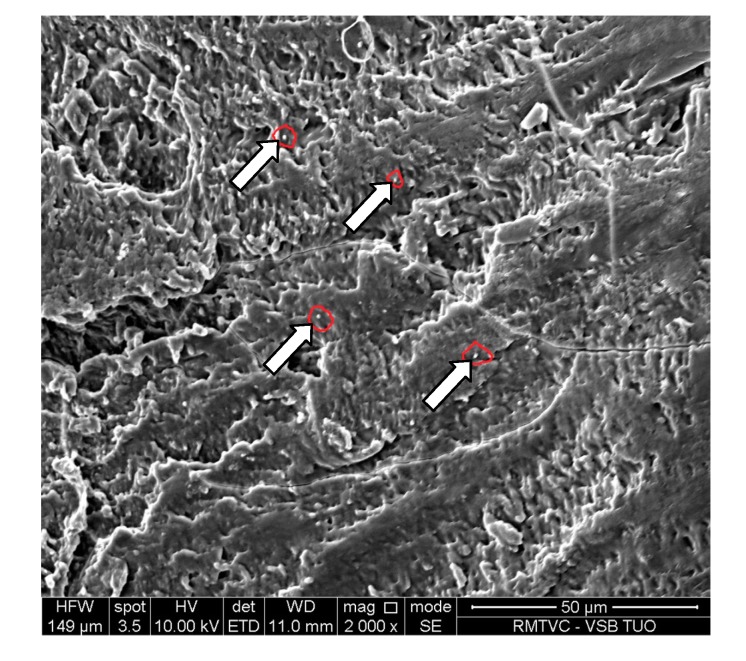
SEM image of risedronate with sodium carboxymethyl dextran (Sample **5c**) at magnification 2000×.

**Figure 11:**
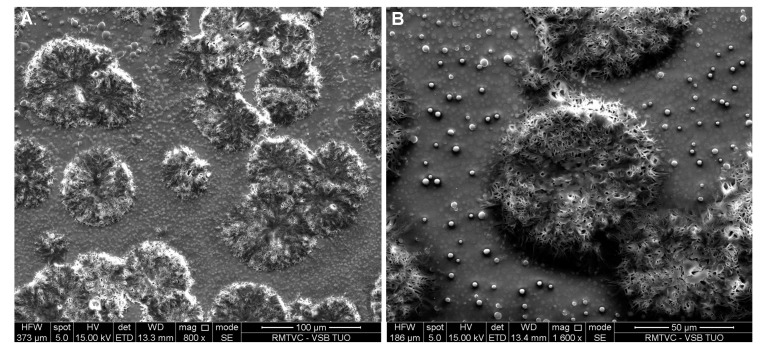
SEM image of risedronate with sodium dodecyl sulphate/macrogol 6000 (Sample **6c**) at magnification 800× (**A**) and 1600× (**B**).

Figure 8, Figure 9, Figure 10 and Figure 11 represent SEM images showing surface structure and morphology of the selected samples at 5% mass concentration of the excipient, in which the occurrence of risedronate nanoparticles was confirmed by dynamic light scattering. The microscopy results confirmed previous results. The occurrence of nanoparticles clusters is clearly visible on Figure 8 representing Sample **2c**. The clusters of risedronate nanoparticles are hidden under the excipient polysorbate 80, which is a viscous excipient that creates coating.

Figure 9A shows spherical particles of risedronate with different sizes anchored in macrogol 6000 matrix; the structure of risedronate with macrogol is shown in Figure 9B in detail. The risedronate nanoparticles placed in polymer matrices are illustrated in Figure 10 and Figure 11. In case of Figure 10 sodium carboxylmethyl dextran forms a layered structure containing spherical nanoparticles of risedronate. In Figure 11 micelles of sodium dodecyl sulphate containing risedronate nanoparticles and risedronate nanoparticles embedded in macrogel 6000 matrix are visible.

The particle size data listed in Table 1 indicate the following relationships of chemical structure and particle size: obviously, the smallest size of particles was observed for hydrophilic non-ionic surfactants–polysorbate 80 (which is sorbitan monooleate with 20 oxyethylene units) and macrogol 6000 (polyethylene glycol). Also, hydrophilic surfactant/viscosity modifier sodium carboxymethyl dextran (which is branched polymeric α-d-glucopyranose substituted by carboxymethyl moieties that are protonated by sodium ions) provides risedronate nanoparticles of the size of few nanometers. All three excipients have ability to generate films and thus coat different small molecules in comparison with sodium dodecyl sulfate, which is an anionic surfactant generating micelles. These results are supported by the fact that stable nanoparticles relatively independent on the excipient concentration were formed by the most effective polymeric “coated” excipients. It is important to note that polysorbate 80, macrogol 6000 and sodium carboxymethyl dextran are soluble in water in contrast to polymeric but poorly water soluble sodium carboxymethyl cellulose (nanoparticles generated only at mass concentration of 1%) that proved to be, due to its aqueous solubility, the least convenient stabilizer of nanoparticles.

Risedronate sodium is a small extremely hydrophilic molecule with a low p*K*_a_ value that gives smaller and more homogenous nanoparticles with polymeric non-ionic stabilizers or with polymeric ionic excipients. Probably due to the character of risedronate this polymeric type of viscous excipients coats and better stabilizes individual particles of risedronate in comparison with the anionic micellar surfactant sodium dodecyl sulfate. This hypothesis is supported by the fact that the particle size of risedronate nanoparticles obtained by combination with sodium dodecyl sulfate and macrogol (3% and 5%) was smaller than that of risedronate nanoparticles with dodecyl sulfate only, see Table 1. Moreover polysorbate 80, macrogol 6000 and sodium carboxymethyl dextran were also found as effective stabilizers for preparation of nanoparticles of steroid compounds, candesartan cilexetil or atorvastatin calcium [23,24].

## 3. Experimental Section

### 3.1. Standardized General Procedure for Preparation of Nanoparticles

Risedronate sodium and all the excipients were purchased from Sigma-Aldrich (St. Louis, MO, USA). All compounds were of analytical grade. H_2_O-HPLC–Milli-Q Grade was used as a solvent of excipients. Each excipient (0.1 g, 0.3 g or 0.5 g) was dissolved in water (10 mL), and three solutions with mass concentrations 1%, 3% and 5% were prepared. Risedronate sodium (0.1 g) was dissolved in water Milli-Q (10 mL), *i.e.*, 1% solutions were prepared. The solution of risedronate in water was slowly dropped (2 mL/min) into the aqueous solutions of excipients that were stirred (600 rpm). Then the system was stirred (600 rpm) for 15 min at 25 °C, after which the mixtures were transferred to an ultrasonic bath in the fume chamber, where they were mixed again for 20 min for homogenization of the sample. Finally the solvent was evaporated.

### 3.2. Particle Size Measurement

The particle size was determined using a Brookhaven dynamic light scattering system BI 9000 (Brookhaven Instruments Corporation, Holtsville, NY, USA) equipped with a SM-200 goniometer and an argon gas laser Lexel 95 (Cambridge Laser Laboratories, Fremont, CA, USA) operating at the wavelength of 514.5 nm. Scattered intensity was registered at the scattering angle 90° and at the temperature of 25 °C. All samples were dispersed by sonication and additionally filtered directly before the measurement through syringe filters with 0.45 μm pore size to remove mechanical impurities. Five independent recordings of the autocorrelation function were done for each investigated excipient concentration. The particle size was calculated from the translational diffusion coefficient using the Stokes-Einstein formula. The translational diffusion coefficient was obtained based on the cumulant expansion of the autocorrelation function up to the second cumulant. The presented particle sizes are reported as the mean values taken of the set of five independent measurements. The results are summarized in Table 1.

### 3.3. FT-IR Analysis

Infrared spectra were recorded by ATR technique with diamond crystal on a Nicolet 6700 FT-IR spectrometer (Thermo Scientific, West Palm Beach, FL, USA). Mid-FT-IR spectra were recorded in the range from 4000 to 400 cm^−1^ with resolution of 4 cm^−1^ and 32 scans. FT-IR spectra were set for chosen samples with different combinations of excipients. The results are illustrated in Figure 2, Figure 3, Figure 4, Figure 5 and Figure 6.

### 3.4. Scanning Electron Microscopy

The morphologies of the samples were examined using a scanning electron microscope (SEM). The samples were attached to microscope glass and then sputtered with gold. The samples were imaged by a scanning electron microscope Quanta FEG 450 (Fei, Hillsboro, OR, USA) with EDS analysis Apollo X (Edax, Weinheim, Germany) using accelerating voltage 10.0 kV, working distance 8.0 mm and probe current 100 pA. The results are illustrated in Figure 8, Figure 9, Figure 10 and Figure 11.

## 4. Conclusions

Eighteen samples of risedronate sodium as a model compound with high solubility and low permeability were prepared by solvent evaporation technique. The common excipients such as sodium dodecyl sulfate, polysorbate 80, macrogol 6000, sodium carboxymethyl cellulose, sodium carboxymethyl dextran and mixture 1:1 of sodium dodecyl sulfate and macrogol 6000 were used as nanoparticle stabilizers. The excipients were applied in mass concentrations of 1%, 3% and 5% in relation to risedronate. The prepared samples were characterized by dynamic light scattering and SEM. FT-MIR spectroscopy was used for verification of sample compositions. Sodium carboxymethyl cellulose in 3% and 5% concentrations provided micro-size Samples (**4b**, **4c**). The particle size of remaining sixteen samples was less than 200 nm, and thirteen samples were under 100 nm. Polysorbate 80, sodium carboxymethyl dextran and macrogol 6000 were determined as the most favourable excipients, particle size of the samples of risedronate with these excipients ranged from 2.8 to 10.5 nm. For these effective nanoparticle stabilizers no influence of the concentration of the excipient on the particle size of risedronate was observed. Based on the results it can be concluded that water soluble polymeric “coated” excipients seem to be more suitable for preparation of risedronate sodium nanoparticles.

## References

[1] Lipinski C.A. (2000). Drug-like properties and the causes of poor solubility and poor permeability. J. Pharmacol. Toxicol. Methods.

[2] Lipinski C.A., Lombardo F. (2001). Experimental and computational approaches to estimated solubility and permeability in drug discovery and development settings. Adv. Drug Deliv. Rev..

[3] Kerns E.H., Li D. (2008). Drug-Like Properties: Concept, Structure Design and Methods.

[4] Sivasankar M., Kumar B.P. (2010). Role of nanoparticles in drug delivery system. Int. J. Res. Pharm. Biol. Sci..

[5] Shaikh M.S., Nikita D. (2012). Permeability enhancement techniques for poorly permeable drugs: A review. J. Appl. Pharm. Sci..

[6] Onoue S., Yamada S., Chan H.K. (2014). Nanodrugs: Pharmacokinetics and safety. Int. J. Nanomed..

[7] Nehoff H., Parayath N.N., Domanovitch L., Taurin S., Greish K. (2014). Nanomedicine for drug targeting: Strategies beyond the enhanced permeability and retention effect. Int. J. Nanomed..

[8] Delie F., Blanco-Prieto M.J. (2005). Polymeric particulates to improve oral bioavailability of peptide drugs. Molecules.

[9] Konan Y.N., Berton M., Gurny R., Allemand E. (2003). Enhanced photodynamic activity of meso-tetra(4-hydroxyphenyl)porphyrin by incorporation into sub-200 nm nanoparticles. Eur. J. Pharm. Sci..

[10] Bawa R. (2008). Nanoparticle-based therapeutics in humans: A survey. Nanotechnol. Law Bus..

[11] Bawa R. (2009). Nanopharmaceuticals for drug delivery–A review. Drug Deliv..

[12] Ezra A., Golomb G. (2000). Administration routes and delivery systems of bisphosphonates for the treatment of bone resorption. Adv. Drug Del. Rev..

[13] Aft R., Perez J.R., Raje N., Hirsh V., Saad F. (2012). Could targeting bone delay cancer progression? Potential mechanisms of action of bisphosphonates. Crit. Rev. Oncol. Hematol..

[14] Van Beek E.R., Lowik C.W., Ebetino F.H., Papapoulos S.E. (1998). Binding and antiresorptive properties of heterocycle-containing bisphosphonate analogs: Structure-activity relationships. Bone.

[15] MedicineNet. http://www.medicinenet.com/risedronate/article.htm.

[16] eMedTV–Health Information Brought to Life™. http://osteoporosis.emedtv.com/.

[17] Risedronate: Drug Bank Online–drugbank.ca. http://www.drugbank.ca/drugs/DB00884.

[18] Mitchel D.Y., Barr W.H., Eusebio R.A., Stevens K.A., Duke F.P., Rusell D.A., Nesbitt J.D., Powell J.A., Thompson G.A. (2001). Risedronate pharmacokinetics intra- and inter-subject variability upon single-dose intravenous and oral administration. Pharm. Res..

[19] Dissette V., Bozzi P., Bignozzi C.A., Dalpiaz A., Ferraro L., Beggiato S., Leo E., Vighi E., Pasti L. (2010). Particulate adducts based on sodium risedronate and titanium dioxide for the bioavailability enhancement of oral administered bisphosphonates. Eur. J. Pharm. Sci..

[20] Oktabec Z., Kos J., Mandelova Z., Havelkova L., Pekarek T., Rezacova A., Placek L., Tkadlecova M., Havlicek J., Dohnal J. (2010). Preparation and properties of new co-crystals of ibandronate with gluco- or galactopyranoside derivatives. Molecules.

[21] Kos J., Pentakova M., Oktabec Z., Krejcik L., Harokova P., Hruskova J., Pekarek T., Dammer O., Havlicek J., Kral V. (2011). Crystallization products of risedronate with carbohydrates and their substituted derivatives. Molecules.

[22] Jung I.W., Han H.K. (2014). Effective mucoadhesive liposomal delivery system for risedronate: Preparation and *in vitro*/*in vivo* characterization. Int. J. Nanomed..

[23] Vaculikova E., Grunwaldova V., Kral V., Dohnal J., Jampilek J. (2012). Primary investigation of the preparation of nanoparticles by precipitation. Molecules.

[24] Vaculikova E., Grunwaldova V., Kral V., Dohnal J., Jampilek J. (2012). Preparation of candesartan and atorvastatin nanoparticles by solvent evaporation. Molecules.

[25] Vaculikova E., Placha D., Cech-Barabaszova K., Jampilek J. (2014). Cimetidine nanoparticles study. Adv. Sci. Eng. Med..

[26] Thorat A.A., Dalvi S.V. (2012). Liquid antisolvent precipitation and stabilization of nanoparticles of poorly water soluble drugs in aqueous suspensions: Recent developments and future perspective. Chem. Eng. J..

[27] U.S. Food and Drug Administration–Generally Recognized as Safe (GRAS). http://www.fda.gov/food/IngredientspackagingLabeling/GRAS/.

[28] Merkus H.G. (2009). Particle Size Measurements: Fundamentals, Practice, Quality.

[29] Win K.Y., Feng S.S. (2005). Effects of particle size and surface coating on cellular uptake of polymeric nanoparticles for oral delivery of anticancer drugs. Biomaterials.

[30] Malvern Instruments Ltd: Dynamic Light Scattering Common Terms Defined. http://www.biophysics.bioc.cam.ac.uk/wp-content/uploads/2011/02/DLS_Terms_defined_Malvern.pdf.

